# Cohesin *RAD21* Gene Promoter Methylation in Patients with Acute Myeloid Leukemia

**DOI:** 10.3390/life14101311

**Published:** 2024-10-16

**Authors:** Kalliopi N. Manola, Sophia Zachaki, Katerina Kakosaiou, Agapi Ioannidou, Marina Kalomoiraki, Theodoros Rampias

**Affiliations:** 1 Laboratory of Health Physics, Radiobiology & Cytogenetics, National Center for Scientific Research (NCSR) “Demokritos”, 15341 Athens, Greece; szachaki@gmail.com (S.Z.); katerinakak@hotmail.com (K.K.); mrshcn@gmail.com (A.I.); mar_ka@ipta.demokritos.gr (M.K.); 2 Biomedical Research Foundation Academy of Athens, 11527 Athens, Greece

**Keywords:** *RAD21*, promoter methylation, acute myeloid leukemia

## Abstract

Background: Aberrant gene promoter methylation is one of the hallmarks of Acute Myeloid Leukemia (AML). *RAD21* is an important gene, implicated in sister chromatids cohesion, DNA repair, the regulation of gene transcription, apoptosis and hematopoiesis. Methods: In this study, we investigate the possible implication of *RAD21* promoter methylation in AML pathogenesis using a cohort of AML patients and a cohort of healthy individuals. Results: *RAD21* promoter methylation was found in 24% of patients and in none of the controls (*p* = 0.023), indicating a possible contribution to AML development. Interestingly, a statistically higher frequency of *RAD21* methylation was observed in patients with trisomy 8 (9/21, 42.9%, *p* = 0.021), while none of the patients with aberrations of chromosome 11 had *RAD21* gene promoter methylation (0%, 0/11, *p* = 0.048). Patients with monosomal and complex karyotypes showed low frequencies of *RAD21* methylation (7.7% and 15.4%, respectively) without reaching statistical significance. Moreover, *ASXL1* mutations were not found to be associated with *RAD21* methylation. Conclusions: This is the first study which provides evidence for a possible pathogenetic role of *RAD21* promoter methylation in AML development and especially in AML with trisomy 8. Further studies of *RAD21* promoter methylation in large series of different AML genetic subgroups may contribute to the elucidation of AML pathogenesis and to the identification of new epigenetic biomarkers with diagnostic and prognostic value.

## 1. Introduction

Acute Myeloid Leukemia (AML), the most common acute leukemia in adults, is characterized by the clonal expansion of leukemic blasts in bone marrow (BM), peripheral blood (PB) and other organs. AML is a heterogeneous disease associated with distinct cytogenetic and molecular alterations, which not only play an essential role in leukemogenesis, but are also important for risk stratification and the treatment selection of AML patients. AML recurrent genetic abnormalities such as t(8;21)(q22;q22.1)/*RUNX1::RUNX1T1*, inv(16)(p13.1q22) or t(16;16)(p13.1;q22)/*CBFB::MYH11*, t(9;11)(p21.3;q23.3)/*MLLT3::KMT2A*, are used to predict survival [1]. Currently, it is commonly believed that the identification of new AML biomarkers will improve patient’s screening, diagnosis, prognosis and the prediction of each patient’s response to treatment, contributing to a better understanding of the molecular basis of this complex disease.

While genetic mutations play a crucial role in AML pathogenesis, epigenetic alterations, particularly aberrant DNA methylation, have emerged as key contributors to the development and progression of this disease. In AML, widespread changes in DNA methylation patterns are frequently observed, leading to the silencing of tumor suppressor genes and the activation of oncogenes. These epigenetic changes can occur through various mechanisms, including mutations in genes encoding epigenetic modifiers (e.g., *DNMT3A, TET2* and *IDH1/2*) and the dysregulation of non-coding RNAs. The identification of specific DNA methylation signatures in AML has the potential to improve prognostication, guide therapeutic decisions and facilitate the development of novel targeted therapies aimed at reversing these epigenetic aberrations [2]. Distinct AML subtypes, defined by specific cytogenetic abnormalities or gene mutations, exhibit unique DNA methylation profiles. AML with t(8;21), inv(16) or t(15;17) translocations display specific methylation signatures that distinguish them from other AML subtypes. Additionally, mutations in genes involved in DNA methylation, such as *DNMT3A* and *TET2*, are associated with distinct methylation patterns and have been used to classify AML into different epigenetic subgroups [3]. The relationship between altered DNA methylation and AML is complex, involving both causative and consequential aspects. Mutations in genes encoding epigenetic regulators, such as *DNMT3A* and *TET2*, are frequently found in AML patients. On the other hand, some DNA methylation changes observed in AML may be a consequence of the disease process itself. The altered cellular environment in AML, including hypoxia and inflammation, can influence DNA methylation patterns. Moreover, the acquisition of additional genetic mutations during leukemic transformation can also contribute to further changes in DNA methylation [2].

The present study investigates the possible involvement of the *RAD21* cohesin gene in AML, for which limited, although interesting, data have been reported. The cohesin complex is a multi-subunit protein assembly that plays a pivotal role in maintaining genomic integrity throughout the cell cycle. *RAD21* is one of the basic subunits of this multi-protein complex that also consists of SMC1A, SMC3 and STAG2 proteins [4]. Its primary function lies in establishing and maintaining sister chromatid cohesion, ensuring the faithful segregation of duplicated chromosomes during mitosis [4,5,6]. Early investigations proposed that the primary consequence of cohesin inactivation was the triggering of aneuploidy [7]. However, recent research has indicated that cohesin inactivation significantly impacts the differentiation of progenitor and stem cells [8]. Furthermore, studies suggest that mutated cohesin proteins can hinder the differentiation of hematopoietic stem and progenitor cells (HSPCs). This occurs through their influence on chromatin accessibility and transcription factor activity, potentially playing a role in the development of leukemia [9].

The cohesin complex influences chromatin architecture, gene expression and the accessibility of DNA to DNA methyltransferases (DNMTs) and other epigenetic modifiers. Recent research has revealed that genes encoding cohesin subunits are frequently affected by somatic mutations in a diverse array of human cancers, notably including myelodysplastic syndrome and AML [10,11,12,13,14,15,16]. These mutations disrupt their normal function in chromatin architecture, leading to altered gene expression [17]. This can affect the expression of *DNMTs*, which are responsible for establishing and maintaining DNA methylation patterns and the expression of other epigenetic factors that modify histones and influence chromatin structure [18]. The dysregulation of DNMTs and epigenetic factors due to cohesin mutations can result in changes in DNA methylation patterns across the genome, contributing to the development and progression of AML [19].

*RAD21* is an important gene located on the 8q24.11 chromosomal region which encodes a DNA double-strand break (DSB) repair protein [6]. It is implicated in sister chromatids cohesion and DNA damage repair, the regulation of gene transcription, the maintenance of nuclear architecture, the biogenesis of centrosomes, meiosis, apoptosis and hematopoiesis [6]. Whole-exome sequencing analysis revealed that *RAD21* knockout in human cell lines results in various chromosomal abnormalities, including translocations, duplications and deletions. Additionally, chromosome fusions are formed and when combined with replication stress, create distant DNA single-ended double-strand ends (DSEs). These DSEs have the potential to trigger harmful rearrangements within the genome [5]. A recent study highlighted that *RAD21* suppresses the self-renewal capacity of blood-forming cells by epigenetically silencing the *HoxA7* and *HoxA9* genes. This finding suggests a potential link between *RAD21* dysfunction and the development of leukemia [20]. Research investigating the methylation patterns of the *RAD21* gene promoter in patients with chronic lymphocytic leukemia (CLL) suggests that this epigenetic modification may play a role in the development of the disease through the promotion of CLL cell self-renewal rather than by causing chromosomal abnormalities [21].

Given that promoter methylation results in gene silencing, the investigation of the methylation status of cohesin genes would be of great importance in AML. Based on the above, in the present research, we investigated the methylation status of *RAD21* gene promoter in a cohort of AML patients. This study is the first one that investigates whether *RAD21* methylation has a possible implication in AML pathogenesis and AML chromosomal abnormalities, correlating the methylation data with patient’s clinicopathological characteristics and cytogenetic profile. 

## 2. Materials and Methods

### 2.1. Patients

Our study cohort comprised 96 AML patients, selected according to their cytogenetic abnormalities, as well as 17 healthy individuals. The diagnosis of AML was established in compliance with the World Health Organization (WHO) classification criteria [22]. Healthy donors were individuals from the general Greek population who were not related to each other, had no history of cancer or abnormal blood cell counts and were recruited the same time. All BM and/or PB samples from patients and healthy donors were immediately sent to the Cytogenetic Laboratory of NCSR “Demokritos” after sample taking.

### 2.2. Cytogenetic Analysis

Cytogenetic analysis was conducted on BM cells collected from all AML patients at the time of diagnosis without prior stimulation. Karyotypic analyses were carried out on trypsin G-banded chromosome preparations. Microscopy and computer imaging techniques (Ikaros, Metasystems GmbH, Altlussheim, Germany) were used to determine the karyotype of each patient. The karyotypes were categorized based on the International System for Human Cytogenetic Nomenclature (ISCN) 2020 guidelines. The analysis was considered successful if either a clonal chromosomal abnormality was identified or at least 20 metaphases were examined.

Complex karyotypes were identified as those with at least three distinct chromosomal abnormalities, excluding cases with recurring genetic abnormalities that define other classes. Additionally, hyperdiploid karyotypes (having an extra copy of three or more chromosomes) without any structural abnormalities were also excluded from the definition of complex karyotypes [1]. 

Monosomal karyotypes (MK) were defined as those having either two or more distinct monosomies (excluding loss of X or Y), or one single autosomal monosomy in combination with at least one structural chromosome abnormality [1].

### 2.3. RAD21 Methylation Detection

To assess RAD21 promoter methylation, genomic DNA was extracted from bone marrow aspirates of AML patients and the peripheral blood/bone marrow of healthy individuals using the QIAamp DNA-extraction midi kit (Qiagen, Hilden, Germany). Extracted DNA was stored at −20 °C for further analysis.

Methylation-Specific PCR (MSP) was employed to quantify the methylation status of the CpG island within the RAD21 promoter region. This technique exploits the differential sensitivity of methylated and unmethylated DNA to specific restriction enzymes. Prior to PCR amplification, genomic DNA underwent enzymatic digestion using the EpiTect Methyl II DNA Restriction Kit (QIAGEN, Hilden, Germany). This kit contains two enzymes: MspI, which cleaves only unmethylated DNA at its recognition sequence (CCGG) within the RAD21 promoter, and HpaII, which cleaves only the methylated form of the same CCGG sequence. This differential digestion allows for the selective amplification of either methylated or unmethylated DNA during the subsequent PCR.

The EpiTect Methyl II restriction digest was set up according to the manufacturer’s protocol. Briefly, 250 ng of DNA was used in each of the following restriction enzyme digest reactions: (1) methylation-sensitive (Ms digest), (2) methylation-dependent (Md digest), (3) mock digest (Mo digest), or (4) methylation-sensitive and methylation-dependent double digest (Msd digest). Following the restriction digestion, the remaining undigested DNA served as a template for qPCR amplification (MSP), using primers specifically designed to target the differentially digested region within the RAD21 promoter CpG island.

MSP was performed using the Epitect Methyl II PCR Assay (QIAGEN) according to the manufacturer’s instructions on a Real-time PCR Biorad CFX96 system (Biorad, Hercules, CA, USA). The PCR reaction mix included the RT2 SYBR Green qPCR Mastermix (330500, Qiagen) and the EpiTect Methyl II PCR primers (335002 EPHS114024-1A, QIAGEN, Hilden, Germany) specifically designed by QIAGEN’s bioinformatics experts to target the differentially digested region within the RAD21 promoter CpG island (SABiosciences CpG island ID: 114024, QIAGEN, Hilden, Germany). The resulting amplification data (Ct values) were analyzed using the EpiTect Methyl II PCR Array data analysis tool (available at https://geneglobe.qiagen.com/rs/product-groups/epitect-methyl-ii-pcr-arrays, accessed on 10 October 2020). This software facilitates the quantification of methylated and unmethylated DNA fractions in each sample.

Based on the manufacturer’s recommendations, and consistent with established thresholds in the literature, samples exhibiting a methylated DNA fraction exceeding 30% of the input DNA were classified as “methylated,” while those with a methylated fraction below 30% were classified as “unmethylated.”

### 2.4. ASXL1 Exon 12 Mutation Analysis

Briefly, the ASXL1 exon 12 was amplified from genomic DNA as described by Gelsi-Boyer and colleagues [23]. The PCR amplifications of bone marrow DNA were performed in a total volume of 25 μL PCR mix containing at least 5 ng template DNA, Taq buffer, 200 pmol of each deoxynucleotide triphosphate, 20 pmol of each primer and 1 unit of Hot Star Taq (Qiagen). The PCR reaction comprised heating at 95 °C for 3 min, followed by 35 cycles of 95 °C for 45 s, 61 °C for 45 s, 72 °C for 1 min and 72 °C for 7 min. The PCR products were purified and directly sequenced using the BigDye Terminator v1.1 cycle sequencing kit (Applied Biosystems, Foster City, CA, USA), including the forward or reverse primer. After G50 purification, sequences were loaded on an ABI 3130XL automat (Applied Biosystems). The sequence data files were analyzed using the Consed version 29.0 (University of Washington, Seattle, WA, USA) within the Phred/Phrap/Consed package and all mutations were confirmed on an independent PCR product. The mutations were confirmed at least twice. Sequencing reactions were performed using the forward primer Fw-ASXL1-Ex12 (5′-AGGTCAGATCACCCAGTCAGTT-3′) and the reverse primer Rev-ASXL1-Ex12 (5′-TAGCCCATCTGTGAGTCCAACTGT-3′).

### 2.5. Statistical Analysis

Chi-square tests were used to investigate associations between the *RAD21* methylation status, demographic features, cytogenetic results and *ASXL1* mutations. Fisher’s Exact Test was employed where appropriate. Statistical significance was set at a *p*-value of less than 0.05. Odds ratios (ORs) were presented with their corresponding 95% confidence intervals (CIs). All statistical analyses were conducted using SPSS version 20 software.

## 3. Results

In the AML group used in our study, the ratio of males to females was 1.09 (50 males/46 females), with an average age of 58.6 years (ranging from 19 to 97 years old). In the control group, the sex ratio was 1.125 (9 males/8 females) and the average age was 50.47 years (ranging from 36 to 92 years old). The sex ratio and the mean age did not significantly differ between the AML group and controls (*p* > 0.05) Out of the 96 AML patients selected, 22 had normal karyotypes and 74 patients had abnormal karyotypes (Supplementary Table S1).

Methylation analysis was successfully conducted on all study participants. No methylation of the *RAD21* gene promoter was detected in healthy donors (methylation < 30%, range: 0.07–8.53%). Specifically, 47% (8/17) of the control group displayed methylation levels less than 1% and 53% of the control group while (9/17) displayed methylation levels in the range of 1–8.53%.

**Figure 1:**
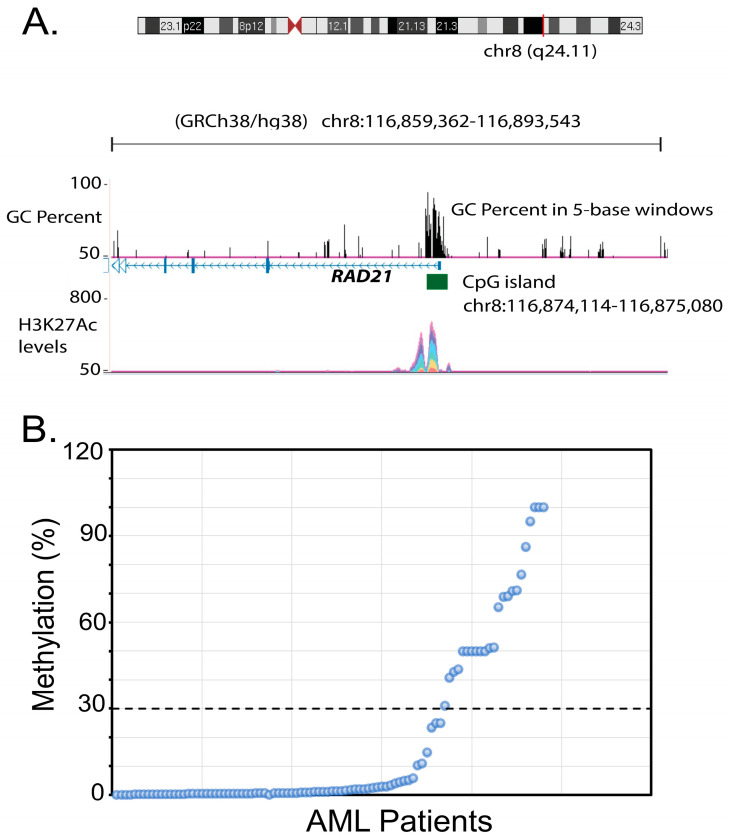
Methylation of *RAD21* gene promoter in AML patients. (**A**) Structure of the *RAD21* gene promoter region indicating the CpG island (1035 bp) and the H3K27Ac peaks based on ENCODE data. (**B**) Methylation levels in the cohort of 96 AML patients based on methylation-specific PCR (MSP) analysis. Samples presenting >30% methylated DNA; fraction of input DNA were defined as methylated in the *RAD21* gene promoter, while those exhibiting <30% were defined as unmethylated based on the instructions of the Epitect Methyl II PCR Assay.

On the contrary, 24% of patients (23/96) were defined as methylated in the *RAD21* gene promoter, presenting methylation levels ≥ 30% of the input DNA (Figure 1A,B; Supplementary Figure S1A,B). Therefore, the frequency of methylated *RAD21* gene promoter was found to be significantly increased in the cohort of AML patients compared to the healthy donors (χ^2^ = 5.114, df = 1, *p* = 0.023) (Table 1).

The proportion of *RAD21* gene promoter methylation was found to vary among patients. As shown in Figure 1B, an unmethylated pattern was detected in 73/96 (76%) of the AML patients, with 45.8% of patients (44/96) displaying 0–1% methylation levels, 23.96% of patients (23/96) displaying 1.1–10% methylation levels and 6.25% of patients (6/96) displaying 10.1–30% methylation levels. The methylated pattern in the *RAD21* gene promoter was detected in 23/96 (24%) of patients, with 18.75% of patients (18/96) displaying 30–80% methylation levels and 5.2% of patients (5/96) displaying 80.1–100% methylation levels. *RAD21* gene promoter methylation was detected in both primary and secondary AML; this was 21.8% (12/55) of de novo AML patients and 27.3% (9/33) of secondary AML patients.

**Figure 2:**
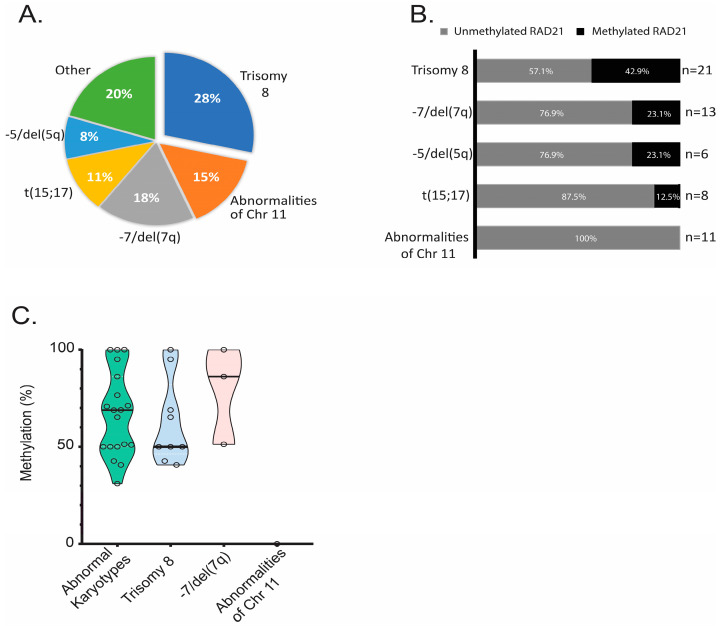
*RAD21* methylation in AML patients with abnormal karyotypes. (**A**) Cytogenetic findings in AML patients with abnormal karyotypes. (**B**) Frequency of *RAD21* methylation in different cytogenetic groups of AML patients. (**C**) Violin plot indicating the *RAD21* methylation levels in methylated samples from different cytogenetic groups of AML patients.

**Figure 3:**
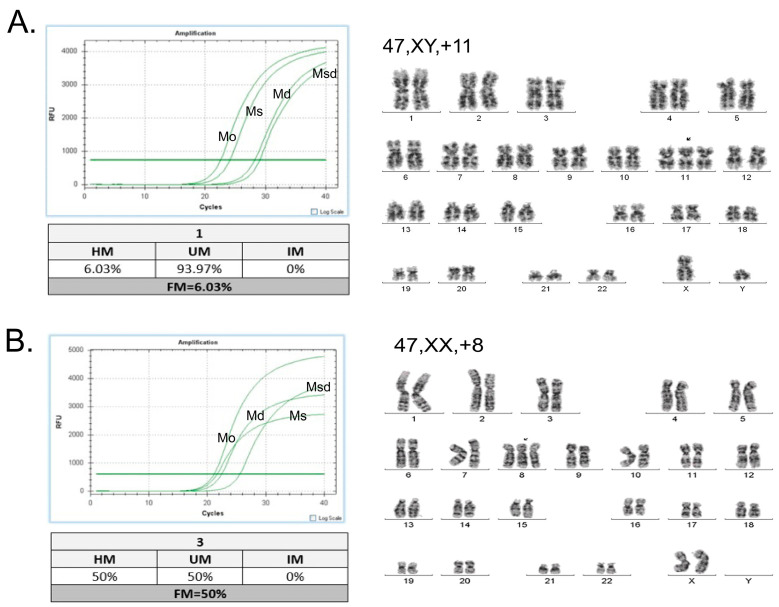
Representative quantitative PCR plots for AML patients that display chr 11 (**A**) and chr 8 (**B**) trisomy. Arrows in karyotypes indicate the extra copy of chromosome 11 or 8.The 4 independent curves in each plot represent the following reactions: (i) reaction after digestion with the methylation-sensitive restriction enzyme (Ms digest); (ii) reaction after digestion with the methylation-dependent restriction enzyme (Md digest); (iii) reaction with undigested template (Mo digest); and finally (iv) reaction after digestion with both the methylation-sensitive and the methylation-dependent restriction enzymes (Msd digest). The relative amount of methylated and unmethylated DNA fractions based on ∆CT values indicate that the *RAD21* gene promoter in the sample with Chr 11 trisomy is unmethylated (FM = HM + IM = 6.03%), while the sample with trisomy 8 is methylated (FM = HM + IM = 50.0%). FM, methylated (M) DNA fraction; HM, hypermethylated; IM, intermediately methylated; UM, unmethylated.

The association of *RAD21* methylation with sex and age revealed no significant differences (Table 1). Concerning sex, 24% of male patients and 23.9% of female patients displayed RAD21 gene promoter methylation. The mean age of patients with *RAD21* gene promoter methylation was 58.3 years versus 58.7 years in those with an unmethylated *RAD21* gene promoter. The stratification of patients in the three age groups revealed similar methylation patterns (Table 1), indicating that *RAD21* methylation is not correlated with the age of diagnosis. The mean blast count was 58.82% in methylated AML patients and 55.61% in unmethylated AML patients (not a statistically significant difference). The stratification of patients, according to the FAB classification based on the type of cell from which the leukemia developed and its degree of maturity, showed that the *RAD21* methylation pattern is more frequent in patients belonging to the M2 subgroup (60%) and less frequent in the M3 subgroup (18.2%). In the next step, to evaluate whether *RAD21* gene promoter methylation is associated with specific cytogenetic abnormalities, the cytogenetic profile and the clinicopathological characteristics of the AML patients were correlated with the methylation data. This analysis showed that methylation was detected in 18.2% of patients with normal karyotypes and in 25.7% of patients with abnormal karyotypes, revealing no statistically significant differences (Table 2). The further stratification of patients according to their karyotypic abnormalities showed that the highest methylation frequency was observed in patients with trisomy 8 (+8) (42.9%, 9/21, *p* = 0.021), while the lowest methylation frequency was observed in patients with abnormalities of chromosome 11 (0/11, 0%, *p* = 0.048) (Figure 2A,B), a cytogenetic group that includes the main structural abnormalities of chromosome 11 that were detected in our cohort of AML patients (Supplementary Table S1). The comparative analysis of *RAD21* gene promoter methylation levels among the different cytogenetic groups revealed that most methylated samples with trisomy 8 display medium-high methylation levels (40–80%) in the *RAD21* gene promoter (Figure 2C). On the contrary, the cytogenetic group of samples with chr 11 abnormalities was strongly associated with the unmethylated status of the *RAD21* gene promoter (Figure 3A,B).

Interestingly, the frequency of patients with *RAD21* gene promoter methylation was quite lower in the group with monosomal karyotypes (7.7%) compared to patients carrying non-monosomal karyotypes; this is probably due to the limited number of patients with monosomal karyotypes. This finding did not reach statistical significance.

Our statistical analysis did not reveal significantly differences in the methylation status between patients with structural chromosomal abnormalities (7/32, 21.9%), numerical chromosomal abnormalities (8/21, 38.1%) or both structural and numerical abnormalities (4/21, 19.1%). In this study, among the eight patients that carried only numerical abnormalities, seven patients had trisomy 8 as a sole aberration and only one patient had -Y as a sole aberration.

Finally, the comparative analysis of the *ASXL1* mutation and *RAD21* methylation data in our cohort of AML patients revealed similar frequencies of methylation between *ASXL1* mutated and *ASXL1* wild-type samples (23.3% and 26.7%, respectively). 

## 4. Discussion

This study is the first to investigate the methylation status of the RAD21 gene in AML patients. The observation that *RAD21* methylation is exclusively detected in AML patients (24%) and not in healthy individuals suggests a potential role for *RAD21* inactivation in AML pathogenesis or as a consequence of the disease. It is well accepted that myeloid malignancy samples exhibiting a reduced expression of cohesin complex genes demonstrate alterations that are analogous to those observed in samples harboring mutations within the cohesin complex [13]. Based on the above, our study presents data supporting that, in addition to mutations in the *RAD21* gene, the methylation of the RAD21 gene promoter constitutes another key mechanism for *RAD21* gene inactivation in AML.

The variation in the methylation levels of the *RAD21* gene promoter among patients could be attributed to the different percentages of AML subpopulations residing in BM tissues and the heterogeneity of AML. An elevated *RAD21* methylation was found in secondary AML patients compared to de novo AML (27.3% vs. 21.8%) but without reaching statistical significance. The evolution of the patients’ DNA methylation status during disease progression is an interesting subject for future research, particularly when long-term patient data are available. The observed frequencies of *RAD21* gene promoter methylation among the different age groups in our cohort of AML patients indicate that *RAD21* gene promoter methylation is not age dependent. The methylation pattern of *RAD21* between normal and abnormal karyotypes showed no statistically significant differences. The lower frequency of RAD21 gene promoter methylation in patients with monosomal karyotypes (7.7%) compared to patients with the abnormal karyotypes (24.3%) may indicate that monosomal karyotypes in AML do not favor *RAD21* gene promoter methylation.

It is worth mentioning that the methylation frequency of *RAD21* was statistically higher in patients with trisomy 8 (+8) (42.9%, 9/21, *p* = 0.021). Trisomy 8, one of the most frequent cytogenetic abnormalities in AML, has been increasingly recognized for its intricate association with altered DNA methylation patterns [24,25,26,27]. A notable observation is the co-occurrence of trisomy 8 with mutations in the genes regulating DNA methylation. Studies have reported a higher frequency of mutations in *DNMT3A*, *TET2*, *IDH1* and *IDH2* in AML patients harboring trisomy 8 compared to those without this cytogenetic aberration [28,29]. Moreover, the additional copies of genes on chromosome 8, including those involved in regulation of DNA methylation (*DNMT3B*, 8p23.1) or in epigenetic regulation (*KAT6A*, 8p11.23; *KMT2B*, 8q24.13; *NSD2*, 8p11.21) may create an environment conducive to the acquisition of DNA methylation, particularly in regions with high DNMT binding affinity or those containing genes sensitive to dosage effects. Hypermethylation is frequently observed on chromosome 8 in AML with trisomy 8, particularly within CpG island shores and interspersed repeats, which can affect the expression of specific genes like *HHEX* [24]. The lack of a global hypermethylation effect on chromosome 8 in trisomy 8 AML suggests that the impact of this chromosomal abnormality on DNA methylation is context-dependent and specific genes or regulatory regions are more susceptible to methylation changes. Since the *RAD21* gene is located on chromosome 8, it is possible that the CpG box in its promoter regulatory regions represents a susceptible region to DNA methylation.

Our findings suggest a potential positive feedback loop in AML, wherein the acquisition of trisomy 8 is associated with the hypermethylation of the *RAD21* gene, a key component of the cohesin complex essential for maintaining chromosomal stability. This, in turn, increases genomic instability, thereby promoting the fitness in AML with trisomy 8. Trisomy 8 is frequently associated with chromosomal abnormalities and complex karyotypes in AML, indicative of high genomic instability [30]. This instability can arise from various mechanisms, including defects in DNA repair pathways, alterations in mitotic checkpoints and the dysregulation of telomere maintenance [31]. The presence of an extra copy of chromosome 8 may further exacerbate this instability by disrupting gene dosage and altering the expression of critical genes involved in DNA repair and chromosome segregation. To this point, genes located on chromosome 8, such as *MYC* and *MOZ*, have been implicated in promoting genomic instability in various cancers. *MYC* overexpression, which is frequently observed in AML, can lead to replication stress and DNA damage [32]. *MOZ*, a histone acetyltransferase, plays a crucial role in regulating gene expression and chromatin structure. Its dysregulation has been linked to chromosomal instability and tumorigenesis [33]. The increased genomic instability in AML with trisomy 8 may facilitate the acquisition of secondary mutations that confer a selective advantage to leukemic cells, fostering tumor heterogeneity and clonal evolution [34]. For example, mutations in *FLT3*, a common driver mutation in AML, are frequently observed in trisomy 8 AML and have been shown to cooperate with trisomy 8 to induce leukemia in mouse models [35]. Studies have demonstrated that AML with trisomy 8 frequently harbors a higher mutational burden compared to other AML subtypes that may contribute to the development of drug resistance, a major challenge in the treatment of AML [36]. To this context, *RAD21* inactivation not only promotes genomic instability but also enhances the tolerance to chromosomal abnormalities in trisomy 8, allowing cells to bypass the normal mitotic checkpoints and survive, despite their abnormal karyotype. Moreover, the absence of *RAD21* gene promoter methylation in all patients with aberrations of chromosome 11 indicates that aberrations of chromosome 11 do not favor *RAD21* gene promoter methylation.

In our study, similar frequencies of *RAD21* methylation were observed between *ASXL1* mutated and *ASXL1* wild-type samples (26.7% and 23.3%, respectively). *ASXL1* mutations has been reported to be associated closely with trisomy 8 (6/18, 33.3%) [37,38]. The elevated frequencies of both *RAD21* gene promoter methylation and *ASXL1* mutations in patients with trisomy 8 compared to other cytogenetic groups, along with the fact that *RAD21* gene is located on chromosome 8, lay the groundwork for further research into this specific cytogenetic group.

In conclusion, this is the first study which provides evidence for RAD21 gene promoter methylation in AML patients. This epigenetic alteration may contribute to the development of AML, especially in AML exhibiting trisomy 8. Larger studies emphasizing AML subtypes according to FAB or WHO classification and AML cytogenetic subgroups are needed to clarify whether *RAD21* methylation status is associated with biologically distinct AML subtypes.

This study provides valuable insights into the epigenetic regulation of the RAD21 promoter in AML. However, we acknowledge some limitations. First, our analysis did not include detailed immunophenotyping and differentiation characterization of the AML samples. Since methylation patterns can be influenced by cellular differentiation, future studies should incorporate detailed immunophenotypic analysis to fully elucidate the relationship between RAD21 methylation and AML differentiation status. Second, this study lacked RAD21 gene expression data. Consequently, while the observed hypermethylation may suggest a potential mechanism for gene silencing, further investigation incorporating gene expression analysis is necessary to elucidate the functional consequences of RAD21 promoter hypermethylation. Finally, we acknowledge that our study had limited data on high-risk cryptic abnormalities (e.g., NUP98::NSD1) and the mutation status of the NPM1, FLT3 and CEBPA genes. These factors could influence RAD21 methylation and should be considered in future studies with more comprehensive genetic data.

The investigation of the methylation status of genes that encode other components of the cohesin complex in AML patients with +8 may help us to identify novel molecular mechanisms that link the epigenetic deregulation with specific cytogenetic alterations in AML pathogenesis. The identification of novel DNA methylation signatures in cohesion complex genes could establish new biomarkers with diagnostic and prognostic value and promote the use of hypomethylating agents as a valuable approach in AML therapy.

## 5. Conclusions

Our findings suggest that the DNA methylation of *RAD21* gene promoter is more frequent in AML patients with trisomy 8. This observation underscores the importance of understanding the complex interplay between genetic and epigenetic alterations in leukemia development. Furthermore, our results highlight the potential of targeting epigenetic modifiers as a therapeutic strategy for AML patients with trisomy 8. By reversing the aberrant DNA methylation patterns, it may be possible to restore *RAD21* expression and improve the clinical outcome of these patients.

## Figures and Tables

**Table 1:** *RAD21* gene promoter methylation results in AML patients and controls. Correlations with demographic characteristics and AML subtypes.

	*n*	Methylated *RAD21* *n* (%)	Unmethylated *RAD21* *n* (%)	*p*-Value
Patients	96	23 (24.0%)	73 (76.0%)	0.023
Controls	17	0 (0.0%)	17 (100.0%)	
Sex				
Male patients	50	12 (24.0%)	38 (76.0%)	ns
Female patients	46	11 (23.9%)	35 (76.1%)	

Blast count (%)	90	58.82%	55.61%	ns
Age subgroups of patients *				
19–45	24	6 (25.0%)	18 (75.0%)	ns
46–64	29	5 (27.8%)	24 (82.8%)	
≥65	41	11 (26.8%)	30 (73.2%)	
FAB subtypes *				
M0	5	1 (20.0%)	4 (80.0%)	ns
M1	5	1 (20.0%)	4 (80.0%)	
M2	10	6 (60.0%)	4 (40.0%)	
M3	11	2 (18.2%)	9 (81.8%)	
M4	13	5 (38.5%)	8 (61.5%)	
M5	8	3 (37.5%)	5 (62.5%)	
M6	0	0 (0.0%)	0 (0.0%)	
M7	1	0 (0.0%)	1 (100.0%)	
De novo and s-AML *				
De novo AML	55	12 (21.8%)	43 (78.2%)	ns
s-AML	33	9 (27.3%)	24 (72.7%)	

* Based on patients with available data. ns: not statistically significant.

**Table 2:** *RAD21* gene promoter methylation results in AML patients in association with cytogenetic and ASXL-1 findings.

	*n*	Methylated *RAD21* *n* (%)	Unmethylated *RAD21* *n* (%)	*p*-Value	
Cytogenetic findings of AML patients					
Normal karyotype	22	4(18.2%)	18 (81.8%)	ns	
Abnormal karyotype	74	19 (25.7%)	55 (74.3%)		
t(9;22)	5	2 (40%)	3 (60%)	ns	
inv(16)/t(16;16)	5	2 (40%)	3 (60%)	ns	
t(8;21)	4	1 (25%)	3 (75%)	ns	
t(15;17)	8	1 (12.5%)	7 (87.5%)	ns	
+8	21	9 (42.9%)	12 (57.1%)	0.021	
−7/del(7q)	13	3 (23.1%)	10 (76.9%)	ns	
Abnormalities of 11	11	0 (0%)	11 (100%)	0.048	
−5/del(5q)	6	1 (16.7%)	5 (83.3%)	ns	
Monosomal karyotypes	13	1 (7.7%)	12 (92.3%)	ns	
Complex karyotypes	13	2 (15.4%)	11 (84.6%)	ns	
ASXL1 mutation status					
ASXL1 unmutated	73	17 (23.3%)	56 (76.7%)	ns	
ASXL1 mutated	15	4 (26.7%)	11 (73.3%)		

ns: not statistically significant

## Data Availability

All data generated or analyzed during this study are included in this article. Further enquiries can be directed to the corresponding authors.

## Supplementary Materials

The following supporting information can be downloaded at https://www.mdpi.com/article/10.3390/life14101311/s1: Figure S1: Genome coordinates of the CpG island on the RAD21 gene promoter. (A) Nucleotide conservation among primates and humans. (B) Nucleotide sequence. Table S1: Abnormal karyotypes according to ISCN 2020 for each of the 74 cases along with mutation status of ASXL-1, prognostic risk group and methylation status.
