# Supplementary material for: Cohesin RAD21 Gene Promoter Methylation in Patients with Acute Myeloid Leukemia
Source: Life (Basel). 2024 Oct 16;14(10):1311. doi: 10.3390/life14101311 (PMC11509327; doi:10.3390/life14101311)
Supplement: Supplementary file 1 [file life-14-01311-s001.zip › Figure S1.pdf]

Supplementary Figure S1

A.

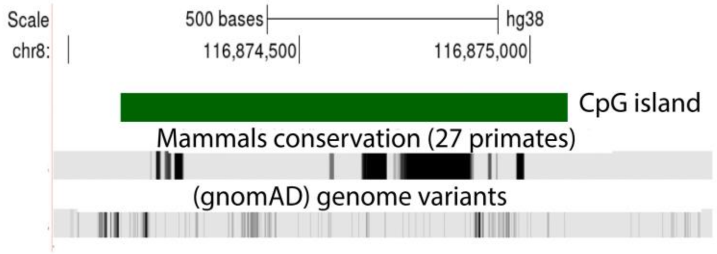

B.

```
>hg38_dna range=chr8:116874114-116875080 strand=+
CGCACCCGAGTTCGGCGGAAGGTTGCTGCTCCCGGGGCTGGCACCGCGGG
CGCCGGGCCCGCCACCGCCTCCTCCCAGACAGCCATTTTTACCCGCGGAG
TAGGCGGCCCGGTCCCCAGAGCGGGCTCGTTCAAACCTCCTCCCCTCCCT
CCGCAGCCCAGGGTTTCCCCGGCCTCCTGGGGGACGTGAGATGGACCTGC
AGGGTAACAGCCTTTGTAGATCTCAGAATGGATCAGAATCATTTGTTACC
GAACAAGCGATGATGCGGGCCCAAGCTGCATGGGTCCGGGCCGAGGGCGG
GCCCGGGGCGAAGGGGGCTGGGCGGGTGGCGCCGCCGCTTGGGCGCCGGG
AGGGTGGCAGCACGCGTGGGCGCGGCGAGGGCTGCCTTCTCCCTCGCCCT
CCCGCCCCCAGGAGTCCGGCTCCCCGACGGCAGAGCGGCGGGGAAAGGGT
GGGGGGAGGGAGCTGGAGGAAAAGAAGGGGTCGGCCGAGTCTCTTACCTT
GCTCTCCGCTGGGAGTTGGGCGGGCTGGGTGGCCCGGGGAGGGGAAAAGG
GTCGGGGGAGGGGGTGGGGAAAGGGGGGAGCCCTTGCGAGGTGTAGCTTC
CGAGCAGCTCCCGCCGCCGCCACAGCCGGCGCCTCCTTTCCGATTCACTC
AAACAAACAAGATGGCTGCCGTTACGCCGCGGCTCTTCCTGCCGCCCAAA
TCCTCGGTTCAAATCGGCAGGATGTTTACGGTCAAAATGGTACCTGTGCG
CCTGCGCAGCCAGCCCAAGCCCCCCCCTCCCCCCAGAAGGAGCGGCGCAG
GCGCAATGACTATTTCCTTTTCTTTGGAACCCGCCCTCTGTTTGTGGAGT
CCACAACTGAGCAAGCGCAAAGGTGATTCTCTTGCGAGGGTCTTTGAGAG
TTGCGGTGTTAGCCAATAGCGTAAGATAGCGCACGCGCAGTGCTTTCCAG
TGCGGTGAATATTTGCG
```

Figure S1. Genome coordinates of the CpG island on the RAD21 gene promoter. (A) Nucleotide conservation among primates and humans. (B) Nucleotide sequence.
