# Supplementary material for: Cohesin RAD21 Gene Promoter Methylation in Patients with Acute Myeloid Leukemia
Source: Life (Basel). 2024 Oct 16;14(10):1311. doi: 10.3390/life14101311 (PMC11509327; doi:10.3390/life14101311)
Supplement: Supplementary file 1 [file life-14-01311-s001.zip › Supplementary table s1.pdf]

Supplementary Table S1: Abnormal karyotypes according to ISCN 2020 for each of the 74 cases along with mutation status of ASXL-1, prognostic risk group and methylation status

**Patient *No***	**Karyotype (ISCN 2020)**	**ASXL1 status**	**Prognostic risk group**	**Methylation status**
1	46,XX,inv(16)(p13q22)[12]/46,XX[8]	wt	F	m
2	44~46,XY,add(1p),-5,-6,-7,-10,-13,-17,-21,+5~7mar,inc[4]/46,XY[16]	wt	A	N
3	43,XX,del(4)(q31),del(5)(q13q33),inv(6)(p23q13),-7,der(12)t(7;12)(p11;p13),-18,-19,-20,+add(22)(p11)[20]	wt	A	m
4	44,XY,del(5)(q13q33),add(6)(p21),-7,-8,-17,-18x2,add(19)(q13.1), -21, +mar1,+mar2,+mar3,+mar4[2]/43~44,XY,add(4)(q31~35), del(5)(q13q33),add(6)(p21),-7,-8,-17,-18x2, add(19)(q13.1), -21,+mar2,+mar3,+mar4,inc[15]	wt	A	N
5	44~45,XY,add(1)(p36.3),-4,-7,-12,-14,-16,del(17)(q21q23), +3~4mar,inc[24]	wt	A	N
6	44~48,X,-X,add(2q),-5,add(7q),del(9q),+del(11)(q23),-14,-15, -17x2,+mar1,+mar2,+mar3,+mar4,inc[10]	wt	A	N
7	45,X,-Y,t(8;21)(q22;q22)[25]	C.1779A<G	A	N
8	45,X,-Y[20]	wt	I	m
9	45,X,-X,t(8;21)(q22;q22)	wt	F	N
10	45,XY,-7,der(18)t(18;?21)(p11.1;q11.2)[20]	wt	A	N
11	45,XX,-9,+mar[6]	wt	A	N
12	45~48,XX,-3,-5,add(6)(p21~23),del(7)(q22),i(8)(q10),-9, inv(9)(p12q13),-17,+20,-21,+1~6mar[13]	wt	A	N
13	46,XX,t(9;22)(q34;q11.2)[18]/46,XX,t(X;12;14)(q13q13q24)[2]/ 46,XX[9]	wt	A	N
14	47,XX,+8[2]/46,XX[20]	wt	I	m
15	46,XX,t(15;17)(q22;q21)[6]/46,XX[5]	wt	F	m
16	47,XY,+8[4]/46,XY[21]	c.1900_1922del	A	N
17	46,XY,inv(16)(p13q22)[18]/46,XY,inv(16)(p13q22),+22[4]/46,XY[3]	wt	F	m
18	46,XY,t(15;17)(q22;q21)[15]/46,XY[5]	wt	F	N
19	45,XX,-7[3]/46,XX [17]	wt	A	N
20	46,XX,inv(12)(p13q13)[13]/46,XX,t(11;12)(q13;p12)[3]/46,XX[9]	wt	I	N
21	46,XY,t(8;16;21)(q22;q24;q22)[16] / 45,X,-Y,t(8;16;21)(q22;q24;q22)[4]	wt	F	N
22	47,XY,+13[12]/48,XY,+13x2[3]/46,XY[15]	wt	I	N
23	46,XY,del(11)(q21q23)[17]/46,XY[3]	wt	I	N
24	46,XY,+8,der(13;13)(q10;q10)[25]	wt	I	N
25	46,XY,der(7)t(1;7)(q11;q11)[22]	wt	I	m
26	46,XY,t(15;17)(q22;q11)[20]	wt	F	N
27	46,XY,t(15;17)(q22;q21)[4]	wt	F	N
28	46,XY,t(8;21)(q22;q22),del(9)(q13q22)[25]	wt	F	N
29	46,XY,t(8;21)(q22;q22)[20]	wt	F	m
30	46,XY,t(9;22)(q34;q11.2)[20]/46,XY[1]	wt	A	N
31	46,XY,del(2p),-4,add(6q),-7,-8,add(11p),+mar1,+mar2,inc[7]/ 46,XY[1]	wt	A	N
32	47,XY,+8[9]/46,XY[11]	wt	I	m
33	47,XY,+8[8]/46,XY[12]	wt	I	N
34	47,XY,+8[7]/46,XY[13]	wt	I	N
35	47,XY,+8[6]/46,XY[14]	c.1934dupG	A	m

36	46,XY,der(18)t(18;21)(p11;q11.2)[5]/46,XY[15]	c.1934dupG	A	m
37	45,XY,del(11)(q13),der(12)t(12;18)(p11.2;q11.2),-18[4]/46,XY[16]	wt	A	N
38	47,XY,+8[2]/46,XY[18]	c.2060G>A	A	N
39	47,XY,+21[7]/46,XY[18]	wt	I	N
40	46,XY,del(20)(q11.2)[18]/46,XY[2]	wt	I	N
41	46,XY,t(9;22)(p24;q11.2)[15]/46,XY[2]	wt	A	N
42	46,XY,inv(16)(p13q22)[27]/46,XY[3]	wt	F	N
43	46,XY,t(9;11)(p22;p15)[20]/46,XY[3]	wt	I	m
44	46,XY,t(15;17)(q22;q21)[16]/46,XY[8]	wt	F	N
45	46,XX,del(5)(q13q33),-17,-18,-21,+mar1,+mar2,+mar3[20]	wt	A	N
46	46,XX,t(15;17)(q22;q21)[25]	wt	F	m
47	46,XX,t(16;16)(p13;q22)[15]/46,XX,inv(2)(p25q11)[6]/ 46,XX,t(7;18)(q31;q23)[3]	wt	I	N
48	46,XX,t(3;3)(q21;q26),t(9;22)(q34;q11.2)[20]	c.1900dupA	A	m
49	46,XX,t(9;22)(q34;q11.2)[20] / 47,XX,+8,t(9;22)(q34;q11.2),i(17)(q10)[4]	N/A	A	m
50	47,XX,+8[19]/46,XX[1]	wt	I	m
51	47,XX,+8[23]/46,XX[1]	N/A	I	N
52	46,XX[10]/46,XX,t(6;9)(p23;q34)[10]	wt	A	m
53	46,XX[18]/46,XX,del(7)(q22)[4]/47,XX,+4[2]	wt	I	N
54	47,XX,+8[7]/46,XX[18]	wt	I	m
55	46,XX,t(15;17)(q22;q21)[7]/46,XX[23]	wt	F	N
56	47,XX,+8[6]/46,XX[24]	wt	I	N
57	46,XX,del(7)(q11.2)[2]/47,XX,+8[2]/46,XX[36]	N/A	I	m
58	46,XX,t(10;13)(q2?6;q14)[26]/46,XX[4]	wt	I	m
59	46,XX,del(11)(q23)[15]/46,XX[5]	wt	I	N
60	47,XX,+8[11]/46,XX[9]	wt	I	m
61	47,XX,+8,t(15;17)(q22;q21)[25]	wt	F	N
62	47,XY,+11[15]	c.1934dupG	A	N
63	47,XY,+11[23]	c.1934dupG	A	N
64	47,XX,+8[24]	c.1934dupG	A	N
65	47,XY,+8[20]	wt	I	m
66	45,XX,-7[5]/46,XX[25]	wt	A	N
67	46,XX,t(15;17)(q22;q21)[7]/46,XX[13]	wt	F	N
68	45,XY,-16,der(17)t(16;17)(p11.1;p11.2)[14]/46,XY,+8,-16, der(17)t(16;17)(p11.1;p11.2)[2]/46,XY[4]	c.1934dupG	A	N
69	46,XX,t(11;17)(q23;q21)[18]/46,XX[2]	wt	A	N
70	45,X,-Y[3]/46,XY[17]	wt	F	N
71	46,XX,t(15;17)(q22;q21)[6]	wt	F	m
72	46,XX,t(9;11)(p22;q23)[13]/46,XX[7]	wt	I	N
73	46,XY,inv(16)(p13q22)[18]/46,XY[2]	wt	F	N
74	47,XX,+14[4]/48,XX,+8,+14[11]/46,XX,del(13)(q12q14)[5]	c.1934dupG	A	N

N/A: not available, wt: wild type, F: favorable, I: intermediate, A: adverse, N:normal, m: methylated
